# Global Trends in Antimicrobial Use in Food Animals from 2017 to 2030

**DOI:** 10.3390/antibiotics9120918

**Published:** 2020-12-17

**Authors:** Katie Tiseo, Laura Huber, Marius Gilbert, Timothy P. Robinson, Thomas P. Van Boeckel

**Affiliations:** 1 Department of Environmental Systems Science, Institute for Environmental Decisions, ETH Zürich, 8006 Zürich, Switzerland; katie.tiseo@usys.ethz.ch (K.T.); laura.huber@usys.ethz.ch (L.H.); 2 Spatial Epidemiology Lab, Université Libre de Bruxelles, 1050 Brussels, Belgium; mgilbert@ulb.ac.be; 3 Fonds National de la Recherche Scientifique, 1050 Brussels, Belgium; 4 Food and Agriculture Organization of the United Nations, 00153 Rome, Italy; timothy.robinson@fao.org; 5 Center for Disease Dynamics Economics and Policy, New Delhi 110024, India

**Keywords:** antibiotics, livestock, animal products, sales, exports

## Abstract

Demand for animal protein is rising globally and has been facilitated by the expansion of intensive farming. However, intensive animal production relies on the regular use of antimicrobials to maintain health and productivity on farms. The routine use of antimicrobials fuels the development of antimicrobial resistance, a growing threat for the health of humans and animals. Monitoring global trends in antimicrobial use is essential to track progress associated with antimicrobial stewardship efforts across regions. We collected antimicrobial sales data for chicken, cattle, and pig systems in 41 countries in 2017 and projected global antimicrobial consumption from 2017 to 2030. We used multivariate regression models and estimated global antimicrobial sales in 2017 at 93,309 tonnes (95% CI: 64,443, 149,886). Globally, sales are expected to rise by 11.5% in 2030 to 104,079 tonnes (95% CI: 69,062, 172,711). All continents are expected to increase their antimicrobial use. Our results show lower global antimicrobial sales in 2030 compared to previous estimates, owing to recent reports of decrease in antimicrobial use, in particular in China, the world’s largest consumer. Countries exporting a large proportion of their production are more likely to report their antimicrobial sales data than countries with small export markets.

## 1. Introduction

Demand for animal protein is increasing globally [1,2,3]. The global expansion of intensive farming has led to an increase in antimicrobial use (AMU) [4] that contributes to the emergence and spread of antimicrobial resistance (AMR) [5]. The World Health Organization defines AMR as “when bacteria, viruses, fungi, and parasites change over time and no longer respond to medicines, making infections harder to treat, and increasing the risk of disease spread, severe illness, and death” [6]. Antimicrobials are an essential component of intensive farming systems and are used to treat and prevent infections, and can also be used in animal feed to increase growth [7,8]. Previous studies have estimated that 73% of all antimicrobials sold globally are used in animals raised for food [9]. AMR in food-producing animals can also affect humans who work closely with animals or live in the vicinity of farms. Food products contaminated with drug-resistant bacteria can also potentially affect the health of humans with AMR pathogens [10,11,12,13,14,15,16]. AMU on farms may also contaminate the environment with drug-resistant pathogens that are potentially harmful to humans [17]. Therefore, monitoring veterinary antimicrobials is essential to curb the rise of AMR, and to track antimicrobial stewardship efforts in humans and animals.

An increased number of surveillance networks for AMR and AMU have been introduced in the last two decades. However, these initiatives have predominantly focused on high income countries, for example, Denmark was the first country to report official antimicrobial sales data and release a report, the Danish Integrated Antimicrobial Resistance Monitoring and Research Programme (DANMAP) in 1996 [18]. In 2011, the European Medicines Agency’s European Surveillance of Veterinary Consumption (ESVAC) group, released their first publication on veterinary antimicrobial sales beginning in 2005 for eight countries (the Czech Republic, Denmark, Finland, France, the Netherlands, Norway, Sweden, and the UK) [19]. Their latest report in 2017 details antimicrobial sales for all countries in the European Union, as well as four additional countries [20]. In North America, Canada began collecting sales data in 2008 for the Canadian Integrated Program for Antimicrobial Resistance Surveillance (CIPARS), which reports on AMR and AMU [21]. In Asia, Japan was the first country to begin a reporting system, the Japanese Veterinary Antimicrobial Monitoring System (JVARM), with data from 2000 [22]. In contrast, in many low- and middle-income countries (LMICs) and developing economies, comparable initiatives have yet to be established.

In countries where the population consumes and imports large quantities of meat, veterinary antimicrobial sales data can help inform consumers about the safety of their food and potentially increase market preference for production systems using less antimicrobials [23,24,25]. Reporting antimicrobial sales data can help guide policymakers in regulating animal product imports coming from countries that use large quantities of antimicrobials and that may contain antimicrobial resistant bacteria and antimicrobial residues. As many countries have their own regulations regarding the amounts and classes of antimicrobials that they can use on domestic animal products [26], some governments may be reluctant to import animal products from countries where AMU is not reported.

As an increasing number of countries report antimicrobial sales data (5 in 2000, 39 in 2015, and 41 in 2017), this offers the opportunity to make statistical inferences on the global trends in veterinary antimicrobial use. Sales data can be interpolated to non-reporting countries using statistical models in combination with information on animal stock and farming systems, or projections for future demand of meat products across regions can be made. In particular, sales reports broken down by species can be used to estimate coefficients of consumption per kilogram of each species of animals farmed around the world. However, cautionary steps must be taken to harmonize antimicrobial sales data and animal population estimates across countries.

This study builds upon reporting efforts from individual countries, and uses statistical models [9] to estimate the total amount of antimicrobials used globally in food-producing animals. We collected and harmonized veterinary antimicrobial sales data from 41 countries to estimate the global consumption of veterinary antimicrobials in 2017. We also projected data for the future food animal populations in each country, and antimicrobial consumption globally in 2030.

## 2. Results

### 2.1. Global Trends in Antimicrobial Use

We estimated that AMU in chicken, cattle, and pigs (which account for 93.75% of all food animals [1]) was 93,309 tonnes of active ingredient (95% CI: 64,443, 149,886) in 2017, and projected an increase of 11.5% by 2030 to 104,079 tonnes (95% CI: 69,062, 172,711) (Supplementary Materials, Figure S1). Pigs had the largest projected increase in antimicrobial consumption and contributed 45% to the total increase between 2017 and 2030. On average, pigs used 193 mg/PCU in 2017. Cattle had the smallest increase in consumption of antimicrobials, accounting for only 22% of the global increase. In 2017, cattle consumed 42 mg/PCU of antimicrobials, the smallest quantity per animal weight of the three food animal groups. Chickens consumed 68 mg/PCU of antimicrobials on average in 2017 and contributed 33% to the global increase in antimicrobial consumption.

**Figure 1:**
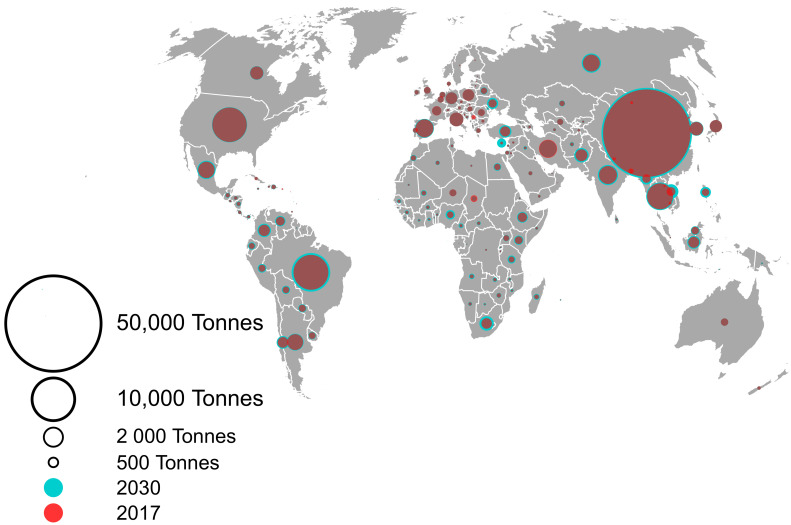
Antimicrobial consumption per country in 2017 and 2030. The size of the circles corresponds to the amounts of antimicrobials used. Dark red circles correspond to the amounts used in 2017, and the outer blue ring corresponds to the projected increase in consumption in 2030.

In both 2017 and 2030, Asia consumed the largest amounts of antimicrobials (57,167 tonnes and 63,062 tonnes, respectively), with an expected increase of 10.3% over this time period. The projected Asian AMU in 2030 amounts to 68% of the antimicrobials used worldwide in 2017. While Africa used lower quantities of antimicrobials in 2017 (4606 tonnes) compared to other regions (Asia, South America, Europe, North America, and Oceania), it has the highest expected increase by 2030 (37%), but this amounts to just 6.1% of the global consumption in 2030 (Figure 1). Oceania, North America, and Europe are expected to have the smallest percentage increase in antimicrobial sales (3.1%, 4.3%, and 6.7%, respectively) (Supplementary Materials Figure S2).

### 2.2. Projected Consumption Increase by Country

**Figure 2:**
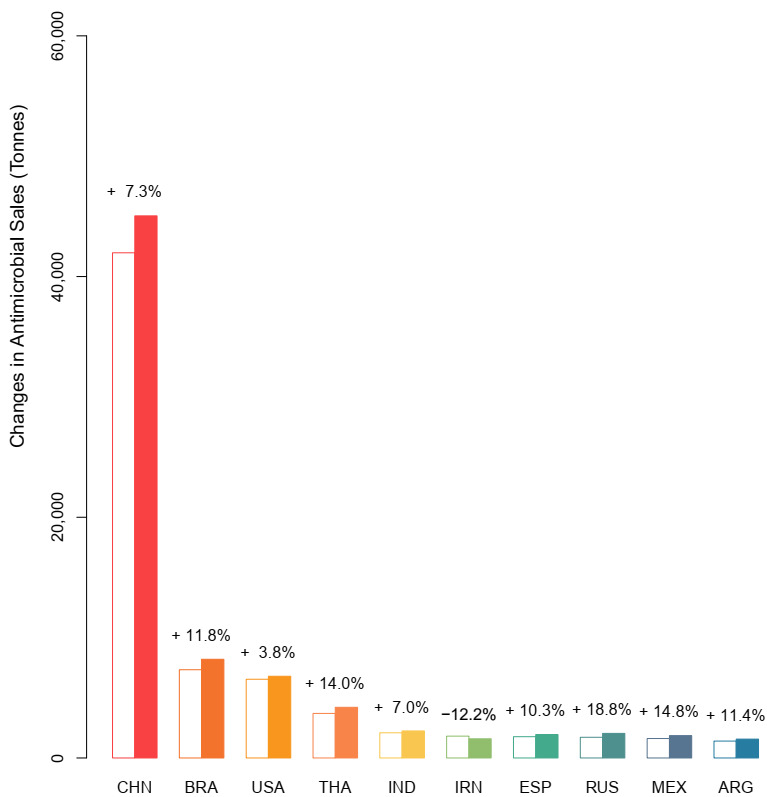
The top 10 consumers of veterinary antimicrobials by country in 2017 (open bars) and their projected consumption for 2030 (closed bars). CHN, China; BR, Brazil; USA, United States; THA, Thailand; IND, India; IRN, Iran; ESP, Spain; RUS, Russia; MEX, Mexico; ARG, Argentina.

In 2017, China was the largest consumer of veterinary antimicrobials, accounting for 45% of global use, and it is projected to remain the largest consumer in 2030 (43%). The top 10 veterinary antimicrobial consumers in 2017 were: China (45%), Brazil (7.9%), the United States (7.0%), Thailand (4.2%), India (2.2%), Iran (1.9%), Spain (1.9%), Russia (1.8%), Mexico (1.7%), and Argentina (1.5%) (Figure 2). Together, these countries account for 75% of the antimicrobials used in animal production, but only 50% of the world’s human population. We estimate that these countries will have increasing use until 2030, with the exception of Iran. While China and Brazil are the top two antimicrobial using countries in 2017, they are not projected to have the largest increases in use compared to the other top 10 consumers. In 2030, these top 10 countries are anticipated to use 72% of the total antimicrobials consumed throughout the world with individual consumption levels estimated as 43% in China, 7.9% in Brazil, 6.5% in the United States, 4.0% in Thailand, 2.1% in India, 1.9% in Spain, 1.9% in Russia, 1.8% in Mexico, 1.5% in Iran, and 1.5% in Argentina.

In China, there are multiple data sources that report highly disparate estimates of the national consumption of antimicrobials. One of these sources is the official government source from the Chinese Ministry of Agriculture for 2017 [27], and another is an estimate from a scientific publication from the Chinese Academy of Sciences [28]. For the present study, we chose to use the official government data, which reported use as 41,967 tonnes. However, the scientific publication estimated that 78,200 tonnes was used, a difference of 86%, which warrants further investigation.

### 2.3. Antimicrobial Sales vs. Meat Exports

**Figure 3:**
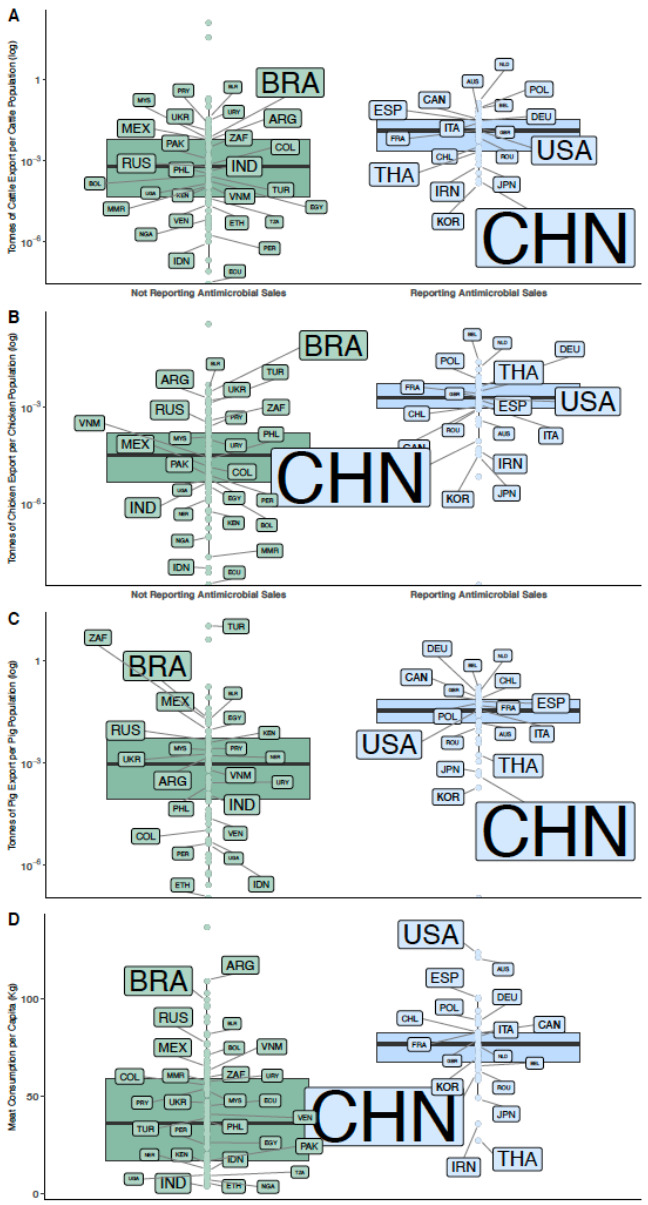
(**A**) Tonnes of cattle exports per cattle population in 2017 in countries reporting antimicrobial consumption (blue) vs. countries that did not report antimicrobial consumption (green); (**B**) Tonnes of chicken exports per chicken population in 2017 in countries reporting antimicrobial consumption (blue) vs. countries that did not report antimicrobial consumption (green); (**C**) Tonnes of pig exports per cattle population in 2017 in countries reporting antimicrobial consumption (blue) vs. countries that did not report antimicrobial consumption (green); (**D**) Meat consumption per capita (in kg) for countries that did and did not report veterinary antimicrobial sales data in 2017. Labels on all boxplots refer to the country’s ISO3 country codes. Size of the ISO3 code is proportional to the amounts of antimicrobials that each country was estimated to use in 2017.

Countries that export a significant share of their animal production (relative to the animal population of each species) were more likely to report antimicrobial sales data than countries where most production is for the domestic market. This was the case for cattle, chicken, and pigs (cattle: Kruskal–Wallis χ^2^ = 33.7, *p* < 0.0001 and Wilcoxon Rank-Sum test ω = 839, *p* < 0.0001; chicken: Kruskal–Wallis χ^2^ = 48.6, *p* < 0.0001 and Wilcoxon Rank-Sum test ω = 545, *p* < 0.0001; pigs: Kruskal–Wallis χ^2^ = 40.6, *p* < 0.0001 and Wilcoxon Rank-Sum test ω = 513, *p* < 0.0001). Reports of antimicrobial sales data were also compared to meat consumption per capita in kilograms (Figure 3). Countries that have a high per capita meat consumption reported antimicrobial sales data more frequently than countries with low meat consumption per capita (Kruskal–Wallis χ^2^ = 43.82, *p* < 0.0001 and Wilcoxon Rank-Sum test ω = 689, *p* < 0.0001). Among reporting countries, the format for reporting varied between countries. Thirty-eight countries reported through official government channels, while one country reported through scientific publications, and two used a combination of both (Supplementary Materials, Table S1). However, non-reporting countries did include notable meat exporters and consumers, such as Brazil, which is one of the top 10 antimicrobial users in 2017 and 2030 (based on statistical estimations).

## 3. Discussion

We estimated that the global consumption of veterinary antimicrobials was 93,309 tonnes in 2017, and projected an increase of 11.5% by 2030 to 104,079 tonnes. It has also been estimated that there will be an increase of 15% for AMU in humans between 2015 and 2030 [29]. This implies that the increasing rate of AMU in humans is consistent with that in food animals. The expected increase in consumption of antimicrobials in animals is less than previous projections. In comparison, Van Boeckel et al. used sales data from 2013, and projected an increase of 53% by 2030 [9]. The discrepancy between past and revised projections stems from multiple sources. First and foremost, highly disparate estimates of antimicrobial sales have been reported from China, which is by far the largest veterinary antimicrobial consumer in the world. These discrepancies have a considerable effect on estimating the global consumption of veterinary antimicrobial sales. In this analysis, we used data from the Chinese Ministry of Agriculture [27]. Previous analyses have used data from a 2015 study by Zhang et al. to estimate AMU in China [28]. The estimates of AMU calculated in the Zhang paper for 2013 was 78,200 tonnes. In contrast, for 2017, the estimate of animal AMU from the Chinese Ministry of Agriculture was 41,967 tonnes. This corresponds to a decrease in consumption of 46.3% over a four-year period. In comparison, the Netherlands, a country that has spearheaded an initiative to reduce its veterinary AMU [30], achieved a reduction of 58% in five years (from 2007–2012) [19,31]. The magnitude of AMU reduction in the Netherlands was less than the decrease in China over a four-year period (38%). The Netherlands were able to achieve a 68% decrease in consumption over a 10-year period using a comprehensive action plan that started in 2008 [30]. China announced its national action plan to combat AMR and AMU in 2016 [32], and one specifically for animals in 2017 [33]. The significant decrease in Chinese veterinary antimicrobial sales from 2013–2017 before the creation of the Chinese national action plan suggests the need for a closer examination of the estimates from these two data sources. Despite this considerable reduction, China would not be the first country to undergo such a dramatic sales decrease in a short amount of time. In Croatia, antimicrobial sales decreased by 47.6% over a three-year period (2014–2017) [20,34]. Another example is Slovenia, where a 52.6% decrease was achieved over three years (2010–2013) [19,35], although their sales increased again in 2014 [34].

A second source of the disparity between past and revised projections for antimicrobials comes from the fact that an increasing number of countries have started reporting antimicrobial sales. These countries include Chile, Croatia, Romania, and Thailand [20,36,37]. Third, in high-income countries (HICs), declines in antimicrobial sales have been reported between 2015 and 2017 in Austria, Belgium, Croatia, Cyprus, Czech Republic, Denmark, Estonia, Finland, France, Germany, Hungary, Iceland, Italy, Latvia, Lithuania, the Netherlands, Slovakia, Spain, Sweden, Switzerland, the United Kingdom, Canada, and the United States (Table 1) [20,38,39,40,41,42]. These decreases are attributable to antimicrobial stewardship efforts in HICs. For example, the United Kingdom decreased antimicrobial use in food animals by 39.2% during this period, which can be attributed to the “the UK Five-Year Antimicrobial Resistance Strategy”, which was published in 2013 [43]. The United States decreased antimicrobial use in animals by 39.66%, and this decrease can be linked to the Guidance for Industry, released in 2012, which advocated for the judicious use of antimicrobials [44]. However, this decrease did not continue in 2018, with a 9% increase between 2017 and 2018 [45]. In Canada, there was a decrease of 21.03% in veterinary antimicrobial sales, which was concomitant with a stewardship program to reduce antimicrobial use in the poultry sector that was originally initiated in 2004 [46]. Finally, we used revised projections of the animal head count from the Food and Agriculture Organization of the UN (see methods) [47,48]. This source uses different methodologies to make their estimates compared to previous reports [49]. The partial equilibrium model used to create new projections has different drivers for agricultural demand and makes different assumptions about forces driving livestock production.

Thus far, no African country has reported veterinary sales data. Over half (52%) of the countries in Sub-Saharan Africa are considered middle-income countries (MICs) and could follow the example of other MICs such as Thailand and China in reporting of veterinary antimicrobial sales. Surveillance systems in Africa are particularly relevant today as the African continent is projected to have the fastest-growing rate of animal protein consumption in the world up to 2027 [50]. There is an increasing number of MICs reporting official government sales data than was the case when estimates were made in previous studies in 2015 and 2017 [4,9] China and Thailand, both considered upper MICs by the World Bank, began reporting their veterinary sales data in publications from 2019 and 2018, respectively. Romania also began reporting its official data in 2014 when it was still categorized as an upper MIC. These examples can help to set precedents for other countries with transitioning livestock sectors and comparable income levels. Similarly, recent work has shown that income is not the sole determinant of the intensity of surveillance of AMR. Multiple low-income countries (LICs) have reported data on AMR rates more frequently than MICs [51] (on a per capita basis). For example, Brazil is an upper MIC that does not report any veterinary antimicrobial sales data although it has a high rate of meat-consumption per capita and exports a large amount of its meat products to other countries. In 2017, Brazil was estimated to have consumed 7.9% of the world’s veterinary antimicrobials, that is, more than France, Germany, Italy, Spain, the United Kingdom, and Poland combined [20].

Reporting of antimicrobial sales is more likely in meat exporting countries than in non-exporting countries (Figure 3). The export of animal products plays an important economic role for many countries [52]. Exports of animal products can be subject to different regulations on AMU compared to those in their home country [26]. Some countries and meat production sectors refuse to import animal products that do not meet national regulations on the maximum concentration of antimicrobial residues [26]. This can result in substantial economic losses or opportunities for a potential exporter [53]. Our analysis shows that countries that report their antimicrobial sales data export a larger proportion of their domestic animal production. Therefore, in the interest of securing export markets, some countries and regions have introduced AMU regulations [26] and have started to report their sales data to improve transparency. However, a notable exception to this trend is Brazil, the world’s largest meat exporter, where antimicrobial sales are not reported. Brazil and other countries that export large amounts of meat that do not report could possibly access larger export markets if they did so. From a consumer standpoint, an increasing number of individuals are becoming aware of the potential benefits of choosing antimicrobial-free products [54,55]. In the United States, consumers have been a driving-force in encouraging companies to reduce the use of growth-promoting antimicrobials in their meat products [23]. Therefore, countries that adopt practices for reducing AMU earlier than others may have an economic advantage to secure export markets.

AMR is a major threat to human health [6], and one of the most important health challenges in the 21st century [56,57]. The overuse of antimicrobials in the animal production sector is a contributor to the increase in AMR. While is clear that AMR in animals is increasing globally [58], ascertaining the health impact on humans from AMU in animals is complex [59,60]. As antimicrobial consumption continues to increase, taking rapid action to report antimicrobial sales data in food-producing animals is essential for AMR surveillance. Countries that do not report their antimicrobial sales data but have the capacity could join a growing group of MICs in documenting global trends in AMU in animals and help promote antimicrobial stewardship globally. Ideally, surveillance systems could be established in cooperation with drug-manufacturers and wholesalers to ensure their accuracy [61]. While systematic surveillance systems may not be established in the short-term in every country, point-prevalence surveys could be a way of monitoring antimicrobial sales in specific geographic areas as a starting point [62]. With a better understanding of where antimicrobial use in food-producing animals may be excessive, countries can initiate stewardship programs, for example, by transitioning to alternative products and promoting better hygiene standards on farms [63].

As with any modeling work, our study comes with limitations. Our study does not take into account the aquaculture sector, which is a rapidly growing source of animal protein [64,65]. However, there is a considerable amount of uncertainty about the amounts of antimicrobials used in this sector, with the exception of salmon [65,66]. This may be especially important in Asian countries where large quantities of seafood are farmed and consumed [52]. Our study is also restricted to sales data from only 41 countries due to a lack of public reports from other countries. However, these 41 countries account for 49.9% of the livestock biomass on earth. Currently, there are no LICs that report antimicrobial sales data. Therefore, data for HICs and MICs were extrapolated to estimate antimicrobial consumption in LMICs. Usage patterns may be different between countries with different income levels. However, we attempted to account for these differences by accounting for the proportion of pigs and chickens raised extensively in LMICs, which is closely correlated with GDP [67]. There may be underestimates in our models due to AMU regulations and other legislation that exists in many HICs and may not exist in LMICs [26]. In addition, the majority of countries that did report AMU did not report sales broken down by animal type. In some countries, the information is not even broken down by classes of antimicrobials (Supplementary Materials, Table S1). Consequently, extrapolations needed to be made, especially for use estimates by animal type. Finally, it is also important to note that antimicrobial sales are only a proxy for actual antimicrobial consumption.

## 4. Materials and Methods

### 4.1. Antimicrobial Sales Data

We conducted systematic online searches for veterinary antimicrobial sales data by country from government sources and from scientific publications [9]. Reports of antimicrobial sales (in kilograms) were collected for 41 countries (Supplementary Materials, Table S1). Only medically important antimicrobials were considered for analysis in this study; coccidiostats and ionophores were excluded [68,69]. Reports of sales were collected using 2017 as a reference, or the most recent year of data available. Data grouped by the class of antimicrobial compound and animal species were used when available. Sales data broken down by at least one animal species was available for 15 countries. All countries reported data broken down by antimicrobial classes, although with varying degrees of detail. However, Iran was the only country that provided pooled data. Sales data for China in 2017 was not separated by any compound classes; however, more recent data from 2018 provided sales broken down by the class of the compounds [27]. We used the percentage of four compound classes (tetracyclines, amphenicols, penicillins, and macrolides/lincosamides) from 2018 and applied these ratios to the sales total from 2017 to make an approximation of Chinese totals by compound class in 2017 and to generate sales data according to the class of the compounds.

### 4.2. Food Animal Census

In this study, we considered three groups of animals raised for food, cattle, chicken, and pigs, which total 93.7% of the animals consumed throughout the world [1]. We estimated the total biomass of animals in each country or region (henceforth referred to as “country”) using population correction units (PCU). The PCU represents the total number of animals in a country (alive or slaughtered), multiplied by the average weight of the animal at the time of treatment. Therefore, the PCU is a standardization metric that takes into account the differences between countries with regard to animal weight and number of production cycles per year. PCUs were calculated with data from the Food and Agriculture Organization of the UN (FAO) projections for the year 2017 [52] using:

$$PCU_{k,s}=An_{k,s}\cdot \left(1+n_{k,s}\right)\cdot \left(\;\frac{Y_{k}}{R_{\frac{\mathrm{CW}}{\mathrm{LW}},k}}\right)$$

where *An_k,s_* is the number of animal type, *k*, for each production system, *s* (either intensive or extensive) in each country; *n_k,s_* is the number of production cycles for each animal type in each production system; *Y_k_* is the quantity of meat in each country for each animal type; and R_CW/LW,k_ is the carcass weight to live weight ratio for each animal type [70]. Data for pigs and chickens were separated by production system estimates [71] and assessed for 2017.

### 4.3. Extrapolation of Consumption

We used a statistical procedure to extrapolate antimicrobial consumption in 187 countries from 41 countries that reported veterinary antimicrobial sales. This five-step statistical procedure is described in Van Boeckel et al. [9] and was revised to include 11 antimicrobial compound classes and three food-animal groups. Coefficients of consumption per PCU were calculated for 11 antimicrobial classes, three animal groups (cattle, chickens, and pigs), 228 countries, and two farming types (intensive or extensive). All statistical analyses were done in R version 3.6.2 [72]. Data cleaning and manipulation was performed using the ‘dplyr’ and ‘tibble’ packages in R [73,74].

### 4.4. Projections for 2017 and 2030

We used the reports of AMU for 2017 to project AMU levels through to 2030 for all 228 countries. First, we calculated the PCU for each country and farming system for 2017 through to 2030. We used three separate datasets for cattle, chicken, and pig populations per country from the FAO publication, *The Future of Food and Agriculture: Alternative Pathways to 2050* [48] for the 1990 through to 2015. These datasets also provided predictions for future populations up to 2050 in five-year intervals. Estimates for the years in between the five-year intervals were calculated using linear regression models. The proportions of chickens and pigs that were raised in extensive and intensive production systems were projected to 2030 using the methodology developed by Gilbert et al. [43,44,45,75] as well as forecasts of GDP per capita (PPP) from the International Monetary Fund (IMF). GDP forecasts from 1980 to 2024 were extended to 2030 using linear regression models. PCU estimates for each country were created by multiplying the estimated animal populations each year by the estimated proportions of animals raised intensively/extensively, and by the estimated mean weight of each animal raised intensively/extensively from 2017 to 2030.

### 4.5. Meat Consumption and Exports

We compared the likelihood of reporting antimicrobial sales data in countries with large and small meat exportation sectors. We collected data on meat consumption per capita, meat exports for cattle, chicken, and pigs, and food animal population for 2017 from FAOSTAT [52]. The availability of export and meat consumption data varied by country and animal species. Meat consumption data were available for 172 countries, while export data for cattle, chicken, and pigs were available for 149, 147, and 139 countries, respectively. Only countries that used more than 175 tonnes of antimicrobials in 2017 were labelled using their ISO3 code (Figure 3). The size of the ISO3 code in each boxplot is proportionate to the total amount of antimicrobials consumed in 2017. Data were checked for normality both visually using a density curve, and with the Shapiro–Wilk test. Statistically significant differences in the distributions (*p* < 0.05) in the boxplots for countries that did and did not report sales data were checked for significance using the non-parametric Kruskal–Wallis and Wilcoxon Rank-Sum tests.

## Figures and Tables

**Table 1:** High-income countries with a reported decrease in total antimicrobial sales from 2015 to 2017. Sales are reported in tonnes.

Country	2015 Sales	2017 Sales (% Decrease) ^1^
Spain	3028.62	1761.68 (−41.83)
United States	10,836.36	6538.52 (−39.66)
The United Kingdom	408.2	248.2 (−39.2)
Slovakia	12.15	8.44 (−30.54)
Croatia	27.87	21.14 (−24.15)
Switzerland	41.34	32.02 (−22.54)
Estonia	8.02	6.27 (−21.82)
Canada	1201.26	948.62 (−21.03)
Italy	1300.24	1057.52 (−18.67)
Hungary	176.18	146.39 (−16.91)
Belgium	258.2	220.99 (−14.41)
Latvia	6.78	5.87 (−13.42)
The Netherlands	214.14	188.42 (−12.01)
Germany	852.49	767.91 (−9.92)
Denmark	102.22	94.08 (−7.96)
Austria	48.47	44.69 (−7.8)
Finland	10.54	9.82 (−6.83)
Czech Republic	47.43	44.23 (−6.75)
France	501.72	483.69 (−3.59)
Cyprus	46.88	45.43 (−3.1)
Lithuania	11.9	11.59 (−2.61)
Iceland	0.58	0.57 (−1.72)
Sweden	10.47	10.31 (−1.53)

^1^ Percent decrease in antimicrobial sales from 2015 to 2017.

## Supplementary Materials

The following are available online at https://www.mdpi.com/2079-6382/9/12/918/s1; Comparison of estimates using 2010 sales data, Figure S1: The estimated global increase of antimicrobial use from 2017 to 2030 by animal type; Figure S2: The estimated increase in antimicrobial use (in tonnes) by continent in 2017 and 2030; Table S1: Antimicrobial sales data sources.
