# Supplementary material for: Global Trends in Antimicrobial Use in Food Animals from 2017 to 2030
Source: Antibiotics (Basel). 2020 Dec 17;9(12):918. doi: 10.3390/antibiotics9120918 (PMC7766021; doi:10.3390/antibiotics9120918)
Supplement: Supplementary file 1 [file antibiotics-09-00918-s001.pdf]

antibiotics

MDPI

Supplementary Materials

Comparisons of Estimates Using 2010 Sales Data

Using antimicrobial sales data from 2010, we previously estimated that 105,596 tonnes of veterinary antimicrobials would be consumed in 2030. In this paper, we estimated that 104,079 tonnes (95% CI: [69,062, 172,711]) of veterinary antimicrobials would be consumed in 2030. This corresponds to an expected consumption decrease of 1.4% in 2030. However, these differences are not significant as the point estimate generated from 2010 sales data is included in our current 95% confidence interval.

In 2010, the five countries with the largest projections of antimicrobial consumption were China (23%), USA (13%), Brazil (9%), India (3%), and Germany (3%). In our current paper, the top five countries for 2017 are China (40%), Brazil (7%), USA (6%), Thailand (4%), and India (2%). While the order of the top five countries has changed along with the overall percentages of the worldwide consumption attributed to them with seven years of new data, four out of the five remain consistent. In our current paper, Germany is no longer in the top five estimated consumers.

In 2010, pigs used the most antimicrobials, averaging at 172 mg/PCU. In 2017, we estimated that pigs averaged 193 mg/PCU. Using this metric, we can see that AMU in pigs is not increasing solely due to an increasing number of pigs, but because more antimicrobials are being used per pig. 2010 sales data indicated that cattle only used 45 mg/PCU of antimicrobials. 2017 sales data indicated that cattle used only 42 mg/PCU, suggesting AMU in cattle may be slowly decreasing. The mean use for chickens in 2010 was 148 mg/PCU. This is much higher than our 2017 predicted mean of 68 mg/PCU. This discrepancy could result from the wide range of AMU in chicken use noted previously [1].

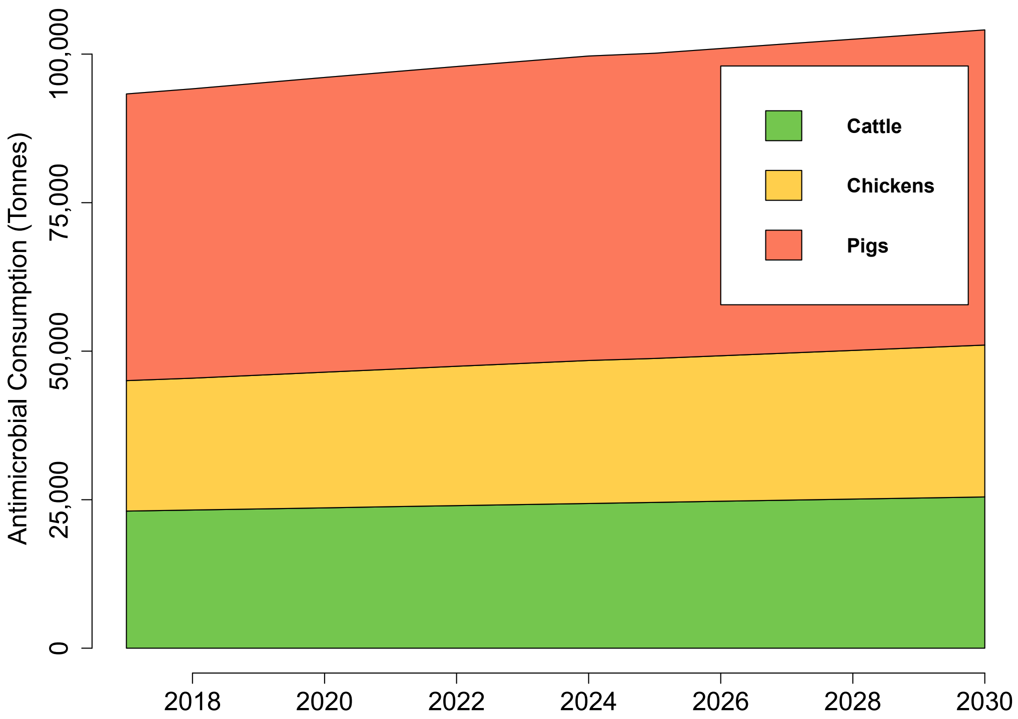

Figure S1. The estimated global increase of antimicrobial use from 2017 to 2030 by animal type.

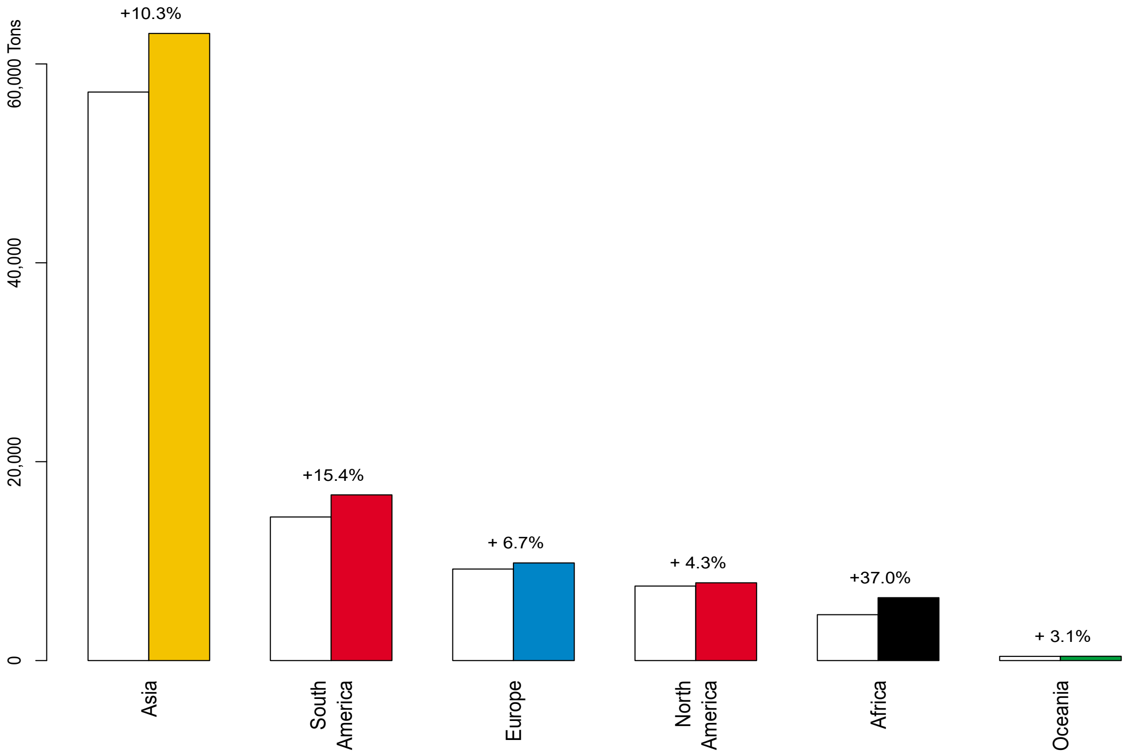

Figure S2. The estimated increase in antimicrobial use (in tonnes) by continent in 2017 and 2030.

Table S1. Antimicrobial sales data sources.

Country	ISO3	Year	Animal Type	Tetracyclines	Amphenicols	Penicillins	Cephalosporins	Sulfonamides	Trimethoprim	Macrolides & Lincosamides	Aminoglycosides	Quinolones	Pleuromutilins	Polymixins	Other Antibiotics	Total Antibiotics	Source
Australia	AUS	2010	Pigs													104200	APVMA
Australia	AUS	2010	Chicken													163100	APVMA
Australia	AUS	2010	Cattle													22510	APVMA
Australia	AUS	2010	All	58000	900	26370	300	13300		71330	5620				126840	305050	APVMA
Austria	AUT	2017	All	24897	382	8204	239	3339	668	3147	1240	477	286	1622	191	44692	EMA
Belgium	BEL	2017	All	56552	3030	83145	505	36860	7406	18682	1010	505	842	3535	8920	220992	EMA
Belgium	BEL	2018	Pigs	28400	1300	46300	10	20800		9300	200	10		2900	1400	110620	BelVet
Belgium	BEL	2018	Chicken	1800	10	10200		3700		7000		390		100		23200	BelVet
Bulgaria	BGR	2017	All	17086	3035	9255	44	2960	412	9817	2286	2323	824	1124	412	49578	EMA
Chile	CHL	2018	All	76661	316582	33068	1306	12182		81587	14633	11295	56521		27464	631299	Agricultural and Livestock Service
China	CHN	2017	All	19262851	2992247	4561812				4079192					11070894	41966996	Ministry of Agriculture

China	CHN	2013	Chicken	1130000		3630000		2450000		2070000		4870000			3970000	18120000	Zhang et al.
China	CHN	2013	Pigs	3300000		10600000		3520000		5840000		13900000			11300000	48460000	Zhang et al.
Croatia	HRV	2017	All	7006	532	6592	118	1774	266	2011	975	769	89	946	59	21137	EMA
Cyprus	CYP	2017	All	18634	1804	8710	46	3372	655	7561	451	301	2513	1117	269	45433	EMA
Canada	CAN	2015	Chicken	25.77		41.85	0	79.49		20.78	3.44	0			274.74	446.07	CIPARS
Canada	CAN	2017	All	501582		107548	6795	61392		140211	11477	640			118971	948616	CIPARS
Czech_Republic	CZE	2017	All	13030	970	11991	555	7555	832	2495	1802	1324	2980	416	277	44227	EMA
Denmark	DNK	2017	All	18941	1439	28531	72	9111	1678	15824	5275	743	8871	480	3117	94082	EMA
Denmark	DNK	2017	Pigs	15212	381	24643	0	6615		13684	6240	0	7669		315	74759	DANMAP
Denmark	DNK	2017	Cattle	1514	737	8163	63	922		240	723	0	0		4	12366	DANMAP
Denmark	DNK	2017	Chicken	483	5	613	0	85		237	65	0	0		0	1488	DANMAP
Estonia	EST	2017	All	1553	55	2407	111	333	67	288	366	144	688	122	133	6267	EMA
Finland	FIN	2013	Chicken													36.5	
Finland	FIN	2017	All	2284	102	4466	20	1827	355	660	51	51	0	0	0	9816	EMA

France	FRA	2017	All	188634	5631	65459	1549	90094	16189	35896	49974	4927	4223	15485	5631	483692	EMA
France	FRA	2017	Cattle				0			0		0				131010	ANSES
France	FRA	2017	Pigs				0			0		0				181270	ANSES
France	FRA	2017	Chicken				0			0		0				96420	ANSES
Germany	DEU	2017	All	198863	5165	297004	4305	61122	7748	66287	16357	9470	16357	73175	12052	767905	EMA
Greece	GRC	2017	All	59282	870	23116	136	10315	1616	4474	5344	8203	1119	1616	621	116712	EMA
Hungary	HUN	2017	All	59899	2236	36001	462	5396	1079	8788	2313	6861	11564	11486	308	146393	EMA
Iceland	ISL	2017	All	38	0	413	1	38	6	0	75	1	0	0	0	572	EMA
Iran	IRN	2010	All													1806896	Aalipour et al.
Iran	IRN	2010	Cattle													1199690	Aalipour et al.
Iran	IRN	2010	Chicken													607207	Aalipour et al.
Ireland	IRL	2015	Cattle	0		925.1	593.9	1.4		4.2	320	0			44.5	1889.1	More et al.
Ireland	IRL	2017	All	41436	2960	20507	845	16701	1268	7399	5285	846			1057	98304	EMA
Italy	ITA	2017	All	304854	23183	271625	2319	130983	15842	165371	42888	22410	35547	20092	22410	1057524	EMA
Japan	JPN	2017	All	347055	27116	99369	3436	98195	13077	165954	44367	7677			36187	842433	JVARM
Japan	JPN	2017	Cattle	19242	3608	16723	2949	9099	956	2016	4457	1336			173	60559	JVARM

Japan	JPN	2017	Pigs	244384	20717	45446	487	65271	10910	77788	29548	1944			35546	532041	JVARM
Japan	JPN	2017	Chicken	22402	1021	21835	0	8534	1152	11550	10083	2952			0	79529	JVARM
Latvia	LVA	2017	All	1463	18	1499	88	194	35	617	617	196	846	229	71	5873	EMA
Lithuania	LTU	2017	All	1665	100	4529	100	2364	533	700	533	269	266	233	300	11592	EMA
Luxembourg	LUX	2017	All	744	44	372	38	312	60	71	55	45	11	11	109	1872	EMA
Korea	KOR	2017	All	254502	114716	280896	11312	88679		107462	50503	47472	24381		23667	1003590	Animal and Plant Quarantine Agency
Korea	KOR	2017	Cattle	16259	2916	43264	4123	2612	317	7124	30	6484	0	1382	100	84611	Animal and Plant Quarantine Agency
Korea	KOR	2017	Pigs	66766	86512	161957	5056	57401	3839	74829	9745	25028	21779	13905	225	527042	Animal and Plant Quarantine Agency
Korea	KOR	2017	Chicken	14179	17929	34625	1792	11655	2072	10998	3403	5092	2602	7129	210	111686	Animal and Plant Quarantine Agency

Malta	MLT	2017	All	183	20	177	4	168	22	181	83	223	407	67	228	1763	EMA
Netherlands	NLD	2017	All	74498	4677	42093	167	28730	5345	25222	1670	3341	1002	1002	668	188415	EMA
Netherlands	NLD	2017	Chicken	2180	0	6225	0	2439		1597	110	1059	30	85	28	13753	SDA
Netherlands	NLD	2017	Pigs	30598	1315	16198	0	13135		7270	32	223	660	767	546	70744	SDA
Netherlands	NLD	2017	Cattle	30824	3460	12891	26	12687		16269	601	1951	0	1108	1090	80907	SDA
New_Zealand	NZL	2008	Cattle	28.7	0	7115.9	1019.2	150.5		53.3	171.8	0.1	0		21.4	8560.8	New Zealand Food Safety Authority
New_Zealand	NZL	2011	Sheep													2500	New Zealand Food Safety Authority
New_Zealand	NZL	2017	All	6821	0	19402	1677	3971		7316	1557	42.5	98	0.4	29529.7	70406	New Zealand Food Safety Authority
Norway	NOR	2017	All	186	372	2978	19	1303	186	37	372	391	37	0	19	5900	EMA
Poland	POL	2017	All	217418	8170	245560	1362	32227	6355	88964	21333	50428	38128	34043	5447	749435	EMA
Portugal	PRT	2017	All	44990	45	35170	701	5812	1202	16842	2705	3617	6313	10922	1703	130022	EMA

Romania	ROU	2017	All	73192	11372	53071	612	7873	1750	42574	31784	13122	13997	11956	1750	263053	EMA
Slovakia	SVK	2017	All	4157	112	2562	180	1348	157	2292	427	768	1191	382	337	13913	SMA
Slovenia	SVN	2017	All	791	129	4083	55	441	110	55	405	533	55	18	37	6712	EMA
Spain	ESP	2017	All	482620	47421	447437	1759	113198	19121	416834	93312	39773	56599	33653	9943	1761670	EMA
Sweden	SWE	2008	Cattle	238	0	746	8	0	6	10	168	20	0		0	1196	SVA National Food Veterinary Institute of Sweden
Sweden	SWE	2008	Pigs	840	0	1218	0	365	72	597	200	18	568		0	3878	SVA National Food Veterinary Institute of Sweden
Sweden	SWE	2008	Chicken	5	0	57	0	0	0	29	0	3	0		0	94	SVA National Food Veterinary Institute of

																Sweden
Sweden	SWE	2017	All	521	21	6580	210	1678	326	514	357	26	78		0	10311	EMA
Switzerland	CHE	2017	All	6851	341	9143	163	10181	591	1574	2462	207		327	181	32021	ARCH-VET
Thailand	THA	2017	All	711473	66	992425	6655	256309	53173	365960	147856	65758	300258	162681	628847	3691461	Ministry of Health
UK	GBR	2017	All	96200		61000	4978	20000	3900	23300	17500	1210			20100	248188	UK Veterinary Medicine Directorate
UK	GBR	2017	Chicken	3300		8200	0	900		1800		38			162	14400	UK Veterinary Medicine Directorate
USA	USA	2017	Cattle	1560542		96936	23512	196902		274479	124675	0			56793	2333839	FDA
USA	USA	2017	Chicken	346597		423689	0	36136		12134	44227	0			76096	938878	FDA
USA	USA	2017	Pigs	1579145		0	0	31024		318145	63602	0			31016	2022932	FDA
USA	USA	2017	All	3535701	49321	690889	29369	274112		621291	259184	22904			1055746	6538517	FDA

Citations

1. Van Boeckel, T.P.; Brower, C.; Gilbert, M.; Grenfell, B.T.; Levin, S.A.; Robinson, T.P.; Teillant, A.; Laxminarayan, R. Global trends in antimicrobial use in food animals. *Proc. Natl. Acad. Sci.* **2015**, *112*, 5649, doi:10.1073/pnas.1503141112.

Publisher's Note: MDPI stays neutral with regard to jurisdictional claims in published maps and institutional affiliations.

© 2020 by the authors. Licensee MDPI, Basel, Switzerland. This article is an open access article distributed under the terms and conditions of the Creative Commons Attribution (CC BY) license (http://creativecommons.org/licenses/by/4.0/).
